# Supplementary material for: Treating Mental Health and Quality of Life in Older Cancer Patients with Cognitive Behavioral Therapy: A Systematic Review and Meta-Analysis
Source: Int J Environ Res Public Health. 2024 Jul 6;21(7):881. doi: 10.3390/ijerph21070881 (PMC11277493; doi:10.3390/ijerph21070881)
Supplement: Supplementary file 1 [file ijerph-21-00881-s001.zip › ijerph-2993792-supplementary.pdf]

Supplemental Table S1. Risk of Bias Assessment

Risk of Bias

	Randomization[1]	Deviation[2]	Missingness[3]	Measurement[4]	Reporting[5]	Overall[6]
Ames et al., 2011	A	A	A	A	A	A
Goode et al., 2011	A	A	B	A	A	A
Johansson et al., 2008	A	A	A	A	A	A
Lau et al., 2020	A	A	A	A	A	A
Mendoza et al., 2017	A	A	B	A	A	A
Mishel et al., 2002	A	A	B	A	A	A
Moon et al., 2020	A	A	A	A	A	A
Mosher et al., 2016	A	A	A	A	A	A
Penedo et al., 2020	A	A	A	A	A	A
Stefanopoulou et al., 2015	A	A	A	A	A	A
van der Meulen et al., 2013	A	A	A	A	A	A
Chambers et al, 2017	A	A	A	A	A	A
Downe-Wamboldt et al, 2007	A	A	A	A	A	A
Graboyes et al, 2022	A	A	A	A	A	A
Mishel et al, 2005	A	A	A	A	A	A
Yanez et al, 2015	A	A	A	A	A	A

* A = Low risk of bias (green color); C = High risk of bias (red color); B = Some concerns (Mid-level risk of bias, yellow color)

1. Risk of bias arising from the randomization process
2. Risk of bias due to deviations from the intended interventions (effect of assignment to intervention)
3. Missing outcome data
4. Risk of bias in measurement of the outcome
5. Risk of bias in selection of the reported results
6. Overall risk of bias

Supplemental Table S2. Risk of Bias Assessment

ROBINS-I

	Confounding[1]	Participant[2]	Classification[3]	Deviation[4]	Missingness[5]	Measurement[6]	Reporting[7]	Overall[8]
Chen et al, 2014	A	B	A	A	A	A	A	A

* A = Low risk of bias (green color); C = High risk of bias (red color); B = Some concerns (Mid-level risk of bias, yellow color)

1. Bias due to confounding
2. Bias in selection of participants into the study
3. Bias in classification of interventions
4. Bias due to deviations from intended interventions
5. Bias due to missing data
6. Bias in measurement of outcomes
7. Bias in selection of the reported result
8. Overall bias
